# Study on the Swelling Characteristics of the Offshore Natural Gas Hydrate Reservoir

**DOI:** 10.3390/e25020278

**Published:** 2023-02-02

**Authors:** Kefeng Yan, Jianyu Zhao, Xiaosen Li, Jingchun Feng, Zhiming Xia, Xuke Ruan

**Affiliations:** 1 Key Laboratory of Gas Hydrate, Guangzhou Institute of Energy Conversion, Chinese Academy of Sciences, Guangzhou 510640, China; 2 Guangdong Provincial Key Laboratory of New and Renewable Energy Research and Development, Guangzhou 510640, China; 3 University of Chinese Academy of Sciences, Beijing 100083, China; 4 Institute of Environmental and Ecological Engineering, Guangdong University of Technology, Guangzhou 510006, China

**Keywords:** natural gas hydrate, porous media, swelling characteristics, structural characteristics

## Abstract

The swelling characteristics of porous media in the offshore natural gas hydrate reservoir have an important effect on the stability of the reservoir. In this work, the physical property and the swelling of porous media in the offshore natural gas hydrate reservoir were measured. The results show that the swelling characteristics of the offshore natural gas hydrate reservoir are influenced by the coupling of the montmorillonite content and the salt ion concentration. The swelling rate of porous media is directly proportionate to water content and the initial porosity, and inversely proportionate to salinity. Compared with water content and salinity, the initial porosity has much obvious influence on the swelling, which the swelling strain of porous media with the initial porosity of 30% is three times more than that of montmorillonite with the initial porosity of 60%. Salt ions mainly affect the swelling of water bound by porous media. Then, the influence mechanism of the swelling characteristics of porous media on the structural characteristics of reservoir was tentatively explored. It can provide a basic date and scientific basis for furthering the mechanical characteristics of the reservoir in the hydrate exploitation in the offshore gas hydrate reservoir.

## 1. Introduction

Natural gas hydrate resources are mainly reserved in permafrost areas and ocean sediments. About 99% of the natural gas hydrate resources are stored in the offshore reservoir [1]. It has been estimated that the natural gas hydrate resources in the South China Sea are equivalent to half of the total proven oil and gas resources in China [2]. Therefore, the research of natural gas hydrate in ocean sediments is significant for the development and utilization of the natural gas hydrate resources.

The natural gas hydrate reservoir is an intricate system. The evolution of the natural gas hydrate reservoir is influenced by the environmental conditions change [3,4,5]. The geological conditions, the gas and water source conditions, the structural characteristics of the porous medium framework, the fluid migration and the reservoir space interact with each other in the natural gas hydrate reservoir. The potential response of the natural gas hydrate reservoir is unknown in the condition of natural or induced external disturbance. The uncertainty of hydrate reservoir performance change exists during the exploitation process. Therefore, it is necessary to study the natural gas hydrate reservoir characteristics and the effect of the hydrate exploitation on the reservoir stability. It is significant for energy utilization and environmental protection. The hydrate reservoirs in permafrost and in offshore have different structural characteristics due to different environmental conditions [3,5,6]. The offshore natural gas hydrate reservoir is composed mainly of argillaceous silty sand in the shallow seabed, including clay, quartz sand, kaolin and other porous media, which are characterized by low permeability and diversity of distribution. The volume swelling and deformation of porous media in the offshore natural gas hydrate reservoir will occur in the condition of external natural or induced external disturbances. The framework of porous media will be deformed, which will lead to the change of the sediment stacking state and the production of sand. In March 2013, a large amount of sand was produced during the 6-day natural gas hydrate testing exploitation in the Coast of Japan, which forced the testing production to be interrupted [7]. In 2017, although the sand control system in China’s testing production of natural gas hydrate in the Shenhu area of the South China Sea was improved, it was still unable to prevent the sand production. At the same time, after the decomposition of the sea mud sample of the testing exploitation, the swelling characteristics of sediments caused by decomposition were observed [8]. Therefore, the swelling characteristics of the reservoir have an important impact on the stability of the offshore reservoir.

Recently, the investigation of the swelling characteristics of porous media mainly focused on the swelling characteristics of the various bentonite. Schanz et al. [9] obtained the linear relationship between swelling pressure and time through the swelling measurement of bentonite. Sun et al. [10] explored the swelling characteristic of bentonite sand mixtures. The results showed the relationship between the pore ratio in a fully saturated state and the swelling pressure were not related with the initial dry density and the initial water content. Xu et al. [11] investigated the swelling deformation characteristics of bentonite in deionized water and salt solution. The results indicated that the type of salt ions and the concentration of salt ions influenced not only the swelling stress of porous media but also the osmotic stress in porous media. At present, there are few studies on the swelling characteristics of the offshore natural gas hydrate reservoir. Li et al. [12,13] explored the process of methane hydrate dissociation in porous media with different swelling characteristics. The investigations found that the porous channel for the flow of gas was blocked due to the formation of bound water in porous media and the change of the swelling characteristics of porous media during the process of methane hydrate dissociation. Then, the permeability of porous media decreased. The cementation between porous media in the sedimentary layer became weak with the shrink of porous medium particles. The porous medium particles fell off from the sedimentary layer, which would affect the multiphase flow of the reservoir [14]. Wang et al. [15] investigated the deformation behavior of porous media during the dissociation of methane hydrate by the depressurization method. The results indicated that the structure of porous media appeared to have the obvious radial shrinkage deformation and the solid migration due to the dissociation of hydrate. It was found that the swelling of porous medium particles changed, which could lead to sand production [16].

The research on the security and stability of natural gas hydrate reservoir during the process of natural gas hydrate exploitation is not systematic and deep due to the complex composition of the offshore natural gas hydrate reservoir in China. It is the key to restricting the development of hydrate commercial exploitation. Therefore, it is urgent to investigate the changes of the hydrate reservoir characteristics during the process of natural gas hydrate exploitation. In this work, we explored the physical characteristics and the swelling characteristics of the human-made porous media and the natural sediments from different sea areas. The effects of the material of porous media, water content, the initial porosity and salinity on swelling characteristics of porous media were discussed. Then, the mechanism of the influence of the porous media swelling characteristics on the structural characteristics of reservoir during the natural gas hydrate dissociation process was tentatively explored. It can provide a basic date and scientific basis for furthering the hydrate reservoir characteristics in the hydrate exploitation in the offshore gas hydrate reservoir.

## 2. Experimental Section

### 2.1. Apparatus and Material

**Figure 1:**
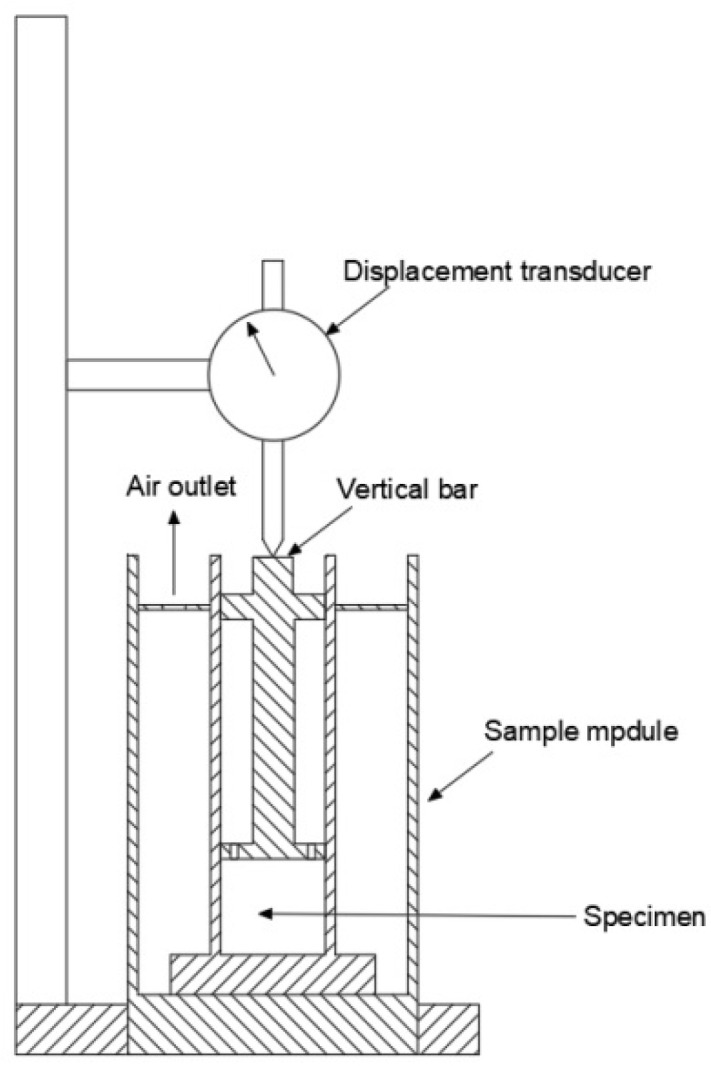
Schematic diagram of dilatometer used in the swelling measurement.

**Figure 2:**
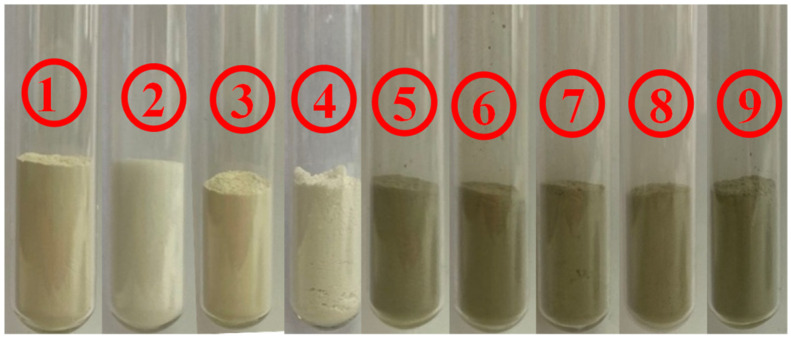
Porous media samples. ① montmorillonite, ② quartz sand (80 mesh), ③ quartz sand (200 mesh), ④ kaolinite, ⑤ NSI, ⑥ NSII, ⑦ NSIII, ⑧ NSIV, ⑨ NSV.

The swellings of the porous media samples were measured by a dilatometer (NP-01) produced by Qingdao Tongchun Oil Instrument Co., Ltd. (Qingdao, China) shown in Figure 1. The porous media samples were montmorillonite, quartz sand, kaolinite and five natural sediments samples from different sea areas, which were collected from different depths below the sea level. The porous media samples and the place of production of each porous media samples are shown in Figure 2 and Table 1. Water was deionized with resistivity of 18.25 mΩ·cm^−1^. NaCl with a purity of 99.8% was supplied by Aladdin Industrial Co., Shanghai, China. The NaCl solutions with different concentrations were prepared.

The densities of the porous media samples were measured by the automatic density analyzer ULTRAPYC 1200e. The particle size distributions and volume weighted mean diameters of the porous media samples were measured by Mastersizer 2000E produced by Malvern Instruments Ltd., Malvern, UK. The elements of the porous media samples were measured by Wavelength Dispersive X-ray Fluorescence Spectrometer (WDXRF) AXIOS mAX petro produced by PANalytical V.B., Almelo, The Netherlands. The specific surface areas were measured by Automated Surface Area and Pore Size Analyzer–Quadrasorb SI produced by Quantachrome Instruments, Boynton Beach, FL, USA. The images of the porous media samples were measured by the scanning electron microscopy (SEM) S-4800 produced by Hitachi, Japan. In the SEM measurement process, the specimens put into the small vessels with inner diameter less than 0.4 mm. Then, the vessels was transferred into the SEM measurement cabin. In the measurement cabin, the vessel was cut and the smooth surface of the specimens in the vessel was exposed for SEM.

### 2.2. Experimental Procedure

In the swelling measurement of the porous media samples, the porous media samples were dried at 110 °C for 24 h in a drying oven and then placed in a sealed container. The dried porous media samples were compacted to a sample module. The dried porous media samples were compacted to a constant height of *H*_1_ using a digital press. When the target height (*H*_1_) was reached, the load was maintained for 1 h for homogenization of the sample. After compaction, the dried porous media samples were placed into a dilatometer and covered with a sealed cap. Deionized water or salt solution was dropped on the dried porous media samples through the center of the sealed cap. The transmission rod was transferred in the variation of the specimens height and the swelling displacement of the specimens was recorded by a strain gauge with a precision of 0.001 mm.

The swelling strain (ε) was defined as the percentage of change in the specimen’s height and was described as:

(1) $$\varepsilon =\frac{\Delta H}{H_{1}}=\frac{H_{2}-H_{1}}{H_{1}}\times 100\%$$

where Δ*H* is the swelling height difference, *H*_1_ is the height of the compacted porous media samples; *H*_2_ is the height of the porous media samples during the swelling process. In this work, when the variation of the swelling strain was less than 0.01% for 3 h, the specimens were considered stabilized.

In this work, the effects of porous material, salinity, water content and the initial porosity on the swelling characteristics of the porous media samples were investigated. The experimental parameters of the swelling characteristics of the porous media samples are listed in Table 2. The initial porosity (ϕ) was calculated as:

(2) $$\phi =\frac{V_{\mathrm{total}}-V_{\mathrm{sample}}}{V_{\mathrm{total}}}\times 100\%=\frac{\pi \times {\left(\frac{d}{2}\right)}^{2}\times H_{1}-\frac{m_{\mathrm{sample}}}{\rho_{\mathrm{sample}}}}{\pi \times {\left(\frac{d}{2}\right)}^{2}\times H_{1}}\times 100\%$$

where *V*_total_ is the volume of the compacted porous media sample, *V*_sample_ is the volume of the dried porous media sample, *d* is the diameter inside the sample maker, *m*_sample_ is the mass of the dried porous media sample, and *ρ*_sample_ is the density of the dried porous media sample, measured by the densitometer.

In the investigation of the physical property of the porous media samples, the surface fractal dimension (*D*) of the porous media samples was calculated. The *D* of porous media is a key factor to test the surface properties of porous media. The calculated methods include the image method, the mercury injection method, the adsorption method, etc. Molecular multilayer adsorption is a method for measuring the *D* of solids by using molecules of a fixed radius to form multilayer adsorption on the surface of a fractal body, and has been widely used in the measurement of the *D* of bentonite. This method uses Frenkel–Halsey–Hill (FHH) equation or Neimark thermodynamic equation to calculate the *D* of porous media. Xiang et al. [17] found that the Neimark method limited to mesoporous where capillary condensation could occur. It was not applicable to smaller interlayer pores. The FHH equation method using full sorption data reflected the actual situation of the entire pore structure, which was more suitable for calculating the *D* of porous media with small pores. Therefore, we used the FHH equation to calculate the *D* of the porous media samples. The FHH equation is expressed as:

(3) $$\ln V_{\mathrm{ads}}=(D-3)\ln\left[\ln(\frac{p_{0}}{p})\right]+\ln C$$

where *V*_ads_ is the volume of gas molecules adsorbed at equilibrium pressure *p*, *p*_0_ is the saturation pressure of the adsorbate, *C* is a constant. *V*_ads_ obtained from the nitrogen adsorption isotherms of the porous media samples measured by Automated Surface Area and Pore Size Analyzer.

## 3. Results and Discussion

### 3.1. Physical Characteristics of Porous Media

The main component of the natural gas hydrate reservoir is argillaceous silty sand, which has the characteristic of low permeability, fine particle and weak cement. The components of the natural gas hydrate reservoir are different with different sea areas, different drilling sits or different collected depths. The elements of the porous media samples were measured by WDXRF. The results show Si and O are the main elements presented in the porous media samples. The results of the elements measurement converted to oxide form and listed in Table 3. It can be seen from Table 3 that the main oxide form of quartz sand is SiO_2_. The main oxide forms of montmorillonite are SiO_2_ and Al_2_O_3_. Montmorillonite is a 2:1 hydrated aluminosilicate, which is described as a tetrahedral octahedral tetrahedral (TOT) layer mineral. Each layer in the montmorillonite structure consists of two outer tetrahedral silicate layers and a central octahedral alumina layer. The molecular formula of montmorillonite is (Al, Mg)_2_[Si_2_O_10_](OH)_2_·nH_2_O. Therefore, the main oxide forms of montmorillonite are SiO_2_ and Al_2_O_3_ corresponding with the analysis result of Table 3.

In Table 3, we can find that the main oxide forms of the natural sediments samples from different sea areas are SiO_2_, Al_2_O_3_ and CaO, which of contents are different in these natural sediments samples. The results are the same as the analysis results of the sediment samples in the drilling sits W01B and W01B of the Shenhu area of South China Sea [18]. The main components of the sediment in the drilling sits W01B and W01B are clay and silty sand. The main composition of clay is montmorillonite and the main composition of the silty sand is SiO_2_. At same time, according to the above analysis, SiO_2_ and Al_2_O_3_ present the main oxide forms of montmorillonite. Therefore, the mole fraction of Al_2_O_3_ could represent the proportion of montmorillonite in the sediments. Moreover, the natural sediments samples collected from different depths below the sea level, which composed some salt ions. In Table 3, the mole fraction of Cl^−^ could represent the proportion of salt ions in the sediments.

**Figure 3:**
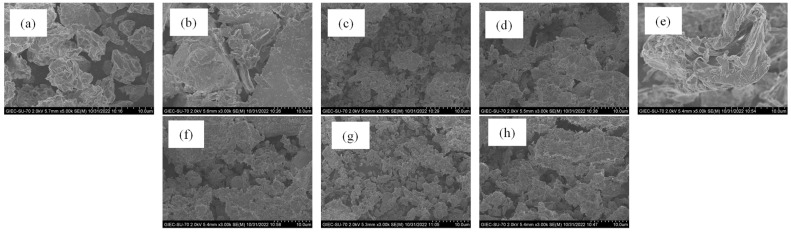
SEM images of porous media samples: (**a**) montmorillonite, (**b**) quartz sand (200 mesh), (**c**) kaolinite, (**d**) NSI, (**e**) NSII, (**f**) NSIII, (**g**) NSIV, (**h**) NSV.

Figure 3 shows the SEM images of the porous media samples. We can find from Figure 3 the surfaces of the porous media samples are rough with sharp edges, which mainly present the flake structure with a large number of voids between the particles. From the images of porous media (Figure 3), the different porous media samples present different surface properties. Each natural sediment sample has the complex internal structure with some small pores with the different geological developments. It is the same as the results of the investigation of the shape factors of the natural sediments in the Shenhu Area of the South China Sea by Shen et al. [19]. It indicated the deeper the buried depth of sediments was, the more complex the structure of sediments. The shape factors of the natural sediments in the Shenhu Area of the South China Sea decreased with the increase in burial depth. Xu et al. [20] proved that the swelling characteristics of porous media were related to the surface properties, especially the fractal dimension. Therefore, we calculated the *D* of the porous media samples to analyze surface properties by the FHH equation, which were listed in Table 4. From Table 4, we can find the particle size distribution scale of the natural sediments samples is μm, which mainly belongs to the silty sand. The *D* is not related to the particle size and the density of the porous media samples. It indicates that the *D* is mainly influenced by the internal pore structure. The *D* will be used as a parameter for the development degree and the internal structure of porous media, which could provide the basic data for the investigation of the physical characteristics of the offshore gas hydrate reservoir.

### 3.2. Swelling Characteristics of Porous Media

#### 3.2.1. Effect of Porous Media Material on the Swelling Characteristics

**Figure 4:**
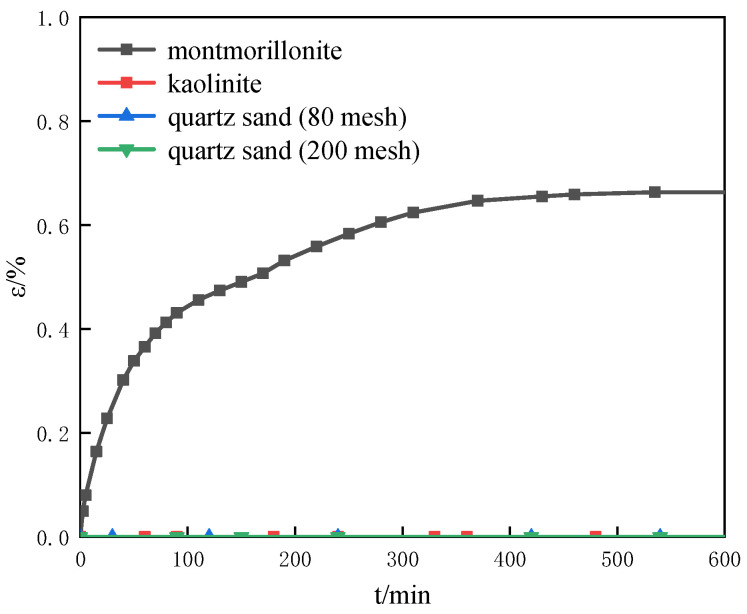
The variation of the swelling strain with time for the different porous media samples with 0 mol/L salinity, 50 wt% water content and 40% the initial porosity.

Figure 4 shows the variation of the swelling strain with time for the different porous media samples with 0 mol/L salinity, 50 wt% water content and 40% the initial porosity. It can be seen from Figure 4 that the swelling strain of montmorillonite has little change (less than 0.01%) after adsorbing water at 400 min indicating the completed swelling. At the same time, it can be seen from Figure 4 that kaolin, quartz sand (80 mesh) and quartz sand (200 mesh) had no swelling after adsorbing water. The results show montmorillonite has a strong capacity to absorb water and has a strong swelling characteristic. It is consistent with previous research [21,22]. The porous material influences the swelling characteristics of the porous media. Therefore, the components of the porous media play an important role in the swelling characteristics of the sediments.

#### 3.2.2. Effect of Water Content on the Swelling Characteristics

**Figure 5:**
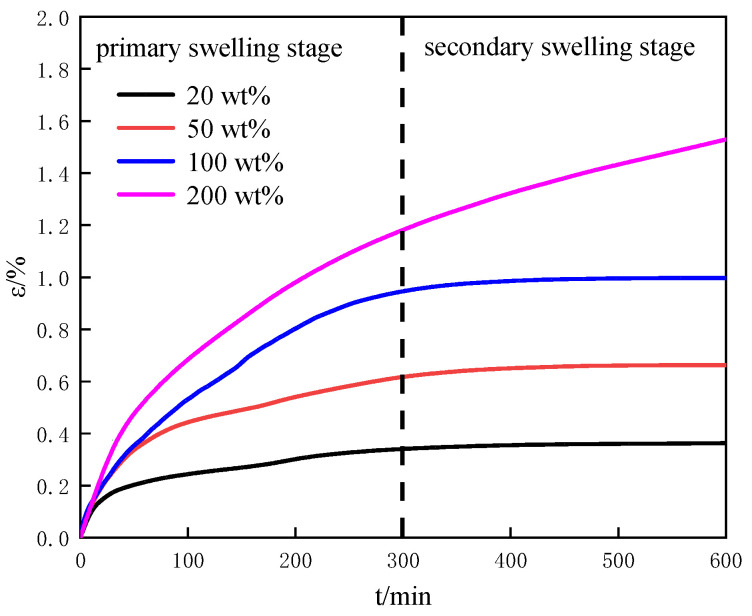
The variation of the swelling strain with time for different water contents at 0 mol/L salinity, 40% the initial porosity.

**Figure 6:**
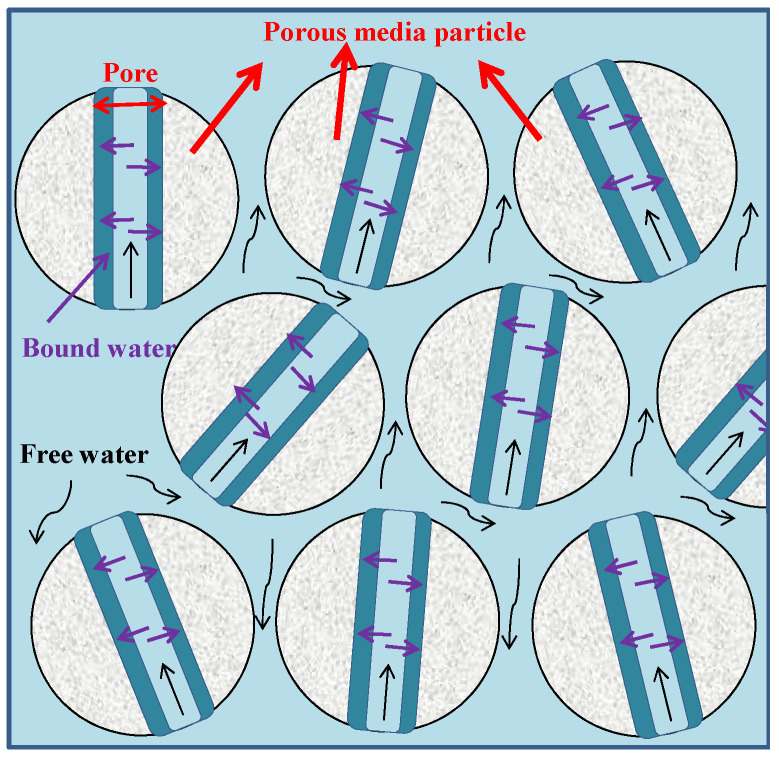
Schematic diagram of liquid flow in porous media. Grey balls indicate the porous media particles; purple arrows indicate the paths of bound water absorbed by porous media; black arrows indicate the paths of free water filled in the space between particles of porous media and the pores of porous media.

We investigated the effect of water content on the swelling characteristics of porous media using the measurement of the swelling of montmorillonite absorbed different water contents due to a strong swelling characteristic of montmorillonite. Figure 5 shows the variation of the swelling strain with time for different water contents at 0 mol/L salinity, 40% the initial porosity. The swelling process divides into two stages due to the different swelling rates: the primary swelling stage and the secondary swelling stage. In the primary swelling stage, montmorillonite absorbs water with rapid swelling. It is the stage in which free water fills the space between particles of montmorillonite and the pores of montmorillonite, which shows in Figure 6. When the pores of montmorillonite fill with free water, montmorillonite swells. It can be seen from Figure 5 that the higher water content is, the faster the swelling rate. When free water fills the pores of montmorillonite, it can be considered as the beginning saturation of water in montmorillonite. The swelling rate reduces. It enters into the secondary swelling stage. It is the stage that bound water is absorbed by montmorillonite, as shown in Figure 6. We find from Figure 5 that the higher water content is, the longer the swelling time is. Therefore, water content has a significant influence on the swelling of porous media. It is consistent with previous research [21,22]. When the expansion time is at 600 min in Figure 5, the swelling strain of montmorillonite with a water content of 200 wt% is about twice that of montmorillonite with a water content of 50 wt%. It reflects the remarkable influence of water content on the swelling again.

#### 3.2.3. Effect of the Initial Porosity on the Swelling Characteristics

**Figure 7:**
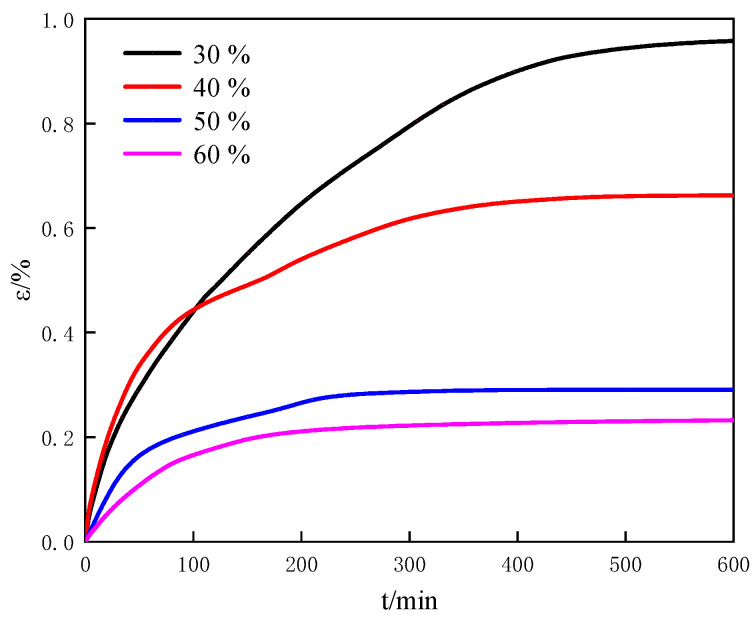
The variation of the swelling strain with time for different initial porosity at 0 mol/L salinity, 50 wt% water content.

The porosities of porous media in the different offshore natural gas hydrate reservoirs are different due to the different stacking structures and the different overburden pressures. The measured porosities of the sediments in the drilling sites W11, W17, W18 and W19 of the South China Sea were 34.5%, 32.2%, 56.7% and 48.3%, respectively [8]. The permeability characteristics of these sediment samples were different with different porosities [8]. Therefore, the porosity of sediments has an important effect on the characteristics of the offshore natural gas hydrate reservoir. We measured the swelling change of montmorillonite with different initial porosities also due to a strong swelling characteristic of montmorillonite. Figure 7 shows the variations of the swelling strain with time for different initial porosity at 0 mol/L salinity, 50 wt% water content. It can be seen from Figure 7 that the swelling rate of montmorillonite decreases as the initial porosity increases. In the primary swelling stage, water firstly fills the space between the particles of montmorillonite, and then fills the pores of montmorillonite, as shown in Figure 6. The larger the initial porosity is, the larger the space between the particles of montmorillonite. In the montmorillonite with the large initial porosity, most of water fills the space between the particles of montmorillonite while less residue water fills the pores of the montmorillonite. Therefore, the swelling strain is smaller in the montmorillonite with the large initial porosity than that in the montmorillonite with the small initial porosity. When the expansion time is at 600 min in Figure 7, the swelling strain of montmorillonite with the initial porosity of 30% is about three times that of montmorillonite with the initial porosity of 60%. Compared with water content, the initial porosity has a more significant influence on the swelling.

#### 3.2.4. Effect of Salinity on the Swelling Characteristics

**Figure 8:**
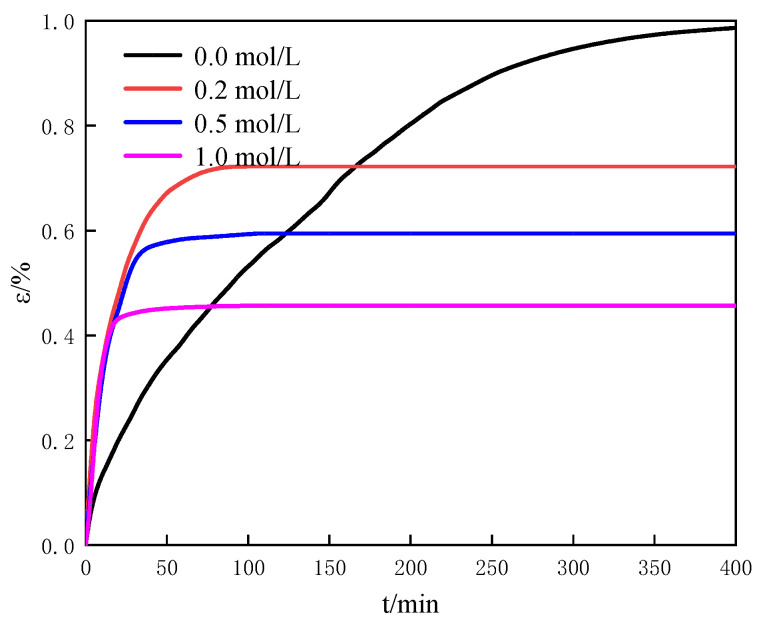
The variation of the swelling strain with time for different salinity at 100 wt% water content, 40% the initial porosity.

In offshore natural gas hydrate reservoir, the formation and exploitation of natural gas hydrate mainly occur in the seawater system. A total of 99% of dissolved matters in the seawater system are salt ions. The electrostatic force of salt ions disrupts the original arrangement of water and forms a hydrated layer around them. The effects of salt ions on the formation and decomposition of the hydrate had been proved in the previous experiments and simulations [23,24]. In this work, the effect of salinity on the swelling characteristics of montmorillonite was investigated by the NaCl solution with different concentrations. Figure 8 shows the variation of the swelling strain with time for different salinities at 100 wt% water content, 40% of the initial porosity. It can be seen from Figure 8 that the swelling montmorillonite decreases as the salt concentration increases. It indicates that salt ions have an inhibitory effect on the swelling montmorillonite, which is the same as previous research [25]. We can find from Figure 8 that the swelling rate of montmorillonite absorbed the NaCl solution is relatively faster than that of montmorillonite absorbed water (salinity = 0 mol/L) in the primary swelling stage. Further, the swelling rates of montmorillonite absorbed the NaCl solutions with different concentrations are approximately the same. In the primary swelling stage, water can quickly enter into the space between particles of montmorillonite and the pores of montmorillonite due to the electrostatic force between salt ions and water. In this stage, the effect of salinity on the swelling montmorillonite is little. In the secondary swelling stage, the swelling montmorillonite decreases as the salt concentration increases. It indicates salt ions mainly influence the swelling of montmorillonite absorbed bound water. When the expansion time is at 400 min in Figure 8, the swelling strain of montmorillonite with salinity of 0.2 mol/L is about one and a half times that of montmorillonite with salinity of 1.0 mol/L. According to the above analysis, it can be concluded that the swelling rate of porous media is directly proportionate to water content and the initial porosity, and inversely proportionate to salinity. Compared with water content and salinity, the initial porosity has much obvious influence on the swelling.

#### 3.2.5. The Swelling Characteristics of the Natural Sediments of the Offshore Natural Gas Hydrate Reservoir

**Figure 9:**
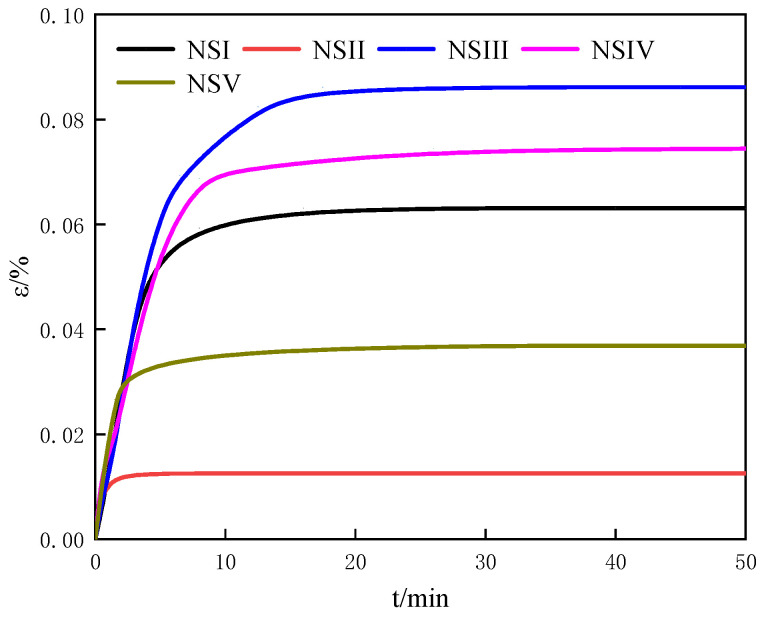
The variation of the swelling strain with time for five natural sediments samples at 0 mol/L salinity, 50 wt% water content, 40% the initial porosity.

Figure 9 shows the variation of the swelling strain with time for five natural sediments samples at 0 mol/L salinity, 50 wt% water content, 40% the initial porosity. As can be seen in Figure 9, the swelling rates of five natural sediment samples are approximately the same in the primary swelling stage, while the swelling rates of five natural sediments samples are different in the secondary swelling stage. As the above analysis states, the mole fraction of Al_2_O_3_ could represent the proportion of montmorillonite in the sediments and the mole fraction of Cl^−^ could represent the proportion of salt ions in the sediments. Montmorillonite has a strong swelling characteristic. Salt ions can inhibit the swelling of porous media. Additionally, the higher concentration is, the more the inhibiting effect of salt ions on the swelling of porous sediment is. Comparing of the swelling of NSI, NSII and NSIII, we can find from Table 3 the mole fractions of Al_2_O_3_ and Cl^−^ in NSII are higher than those in NSI and NSIII. The mole fraction of Cl^−^ in NSIII is lower than that in NSI and NSII. It can be seen from Figure 9 in the secondary swelling stage that the swelling strain of NSIII is highest of NSI, NSII and NSIII. The swelling strain of NSII is the lowest of NSI, NSII and NSIII. It indicates the effect of the content of salt ions on the swelling of porous media is more than the effect of the content of montmorillonite. At the same time, we can find from Table 3 the mole fractions of Al_2_O_3_ and Cl^−^ in NSV are higher than those in NSIII and NSIV. The mole fraction of Cl^−^ in NSIII is lower than that in NSIV and NSV. It can be seen from Figure 9 in the secondary swelling stage that the swelling strain of NSIII is highest of NSIII, NSIV and NSV. The swelling strain of NSV is the lowest of NSIII, NSIV and NSV. It also indicates the effect of the content of salt ions on the swelling of porous media is more than the effect of the content of montmorillonite. However, we also find the swelling strain of NSV is higher than that of NSII, while the mole fractions of Al_2_O_3_ and Cl^−^ in NSV are higher than those in NSII. Therefore, the swelling characteristics of the natural sediments samples is influenced by the coupling of the content of montmorillonite and the content of salt ions. The content of salt ions may be the main factor of influence on the swelling of porous media. However, it is difficult to determine the proportional relationship between the effect of montmorillonite content and salt ion content on the swelling characteristics due to the complex component of the natural sediments.

#### 3.2.6. A Tentative Exploration on the Influence Mechanism of the Swelling Characteristics of Porous Media on the Structural Characteristics of Reservoir

**Figure 10:**
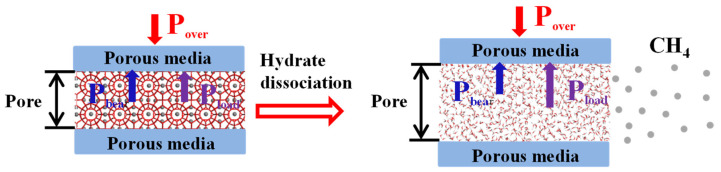
Schematic diagram of the force variation on reservoir during natural gas hydrate exploitation.

Tanikawa et al. [26] found porosity and permeability decreased as the effective stress increased by exploring the core samples of the natural gas hydrate reservoirs. Therefore, this effective stress would have influence on structural characteristics of the natural sediments. This effective stress would be the overburden pressure (P_over_) of the natural gas reservoir. In the natural gas reservoir, there is a balanced relationship among the P_over_, the bearing capacity (P_bear_) of the porous media skeleton and the vertical load force (P_load_) of the porous media swelling as shown in Figure 10. It was defined as follows:

(4) $$P_{\mathrm{over}}=P_{\mathrm{bear}}+P_{\mathrm{load}}$$

P_bear_ is related to the structural characteristics of porous media. And P_load_ is related to the swelling characteristics of porous media. Figure 10 shows the schematic force change of the exploitation of natural gas hydrate in the offshore natural gas hydrate reservoir. It can be seen from Figure 10 that methane overflows from natural gas hydrate after the hydrate dissociation. Water which is produced by the hydrate dissociation would cause the salt dilution effect [27,28]. The swelling force of porous media increases due to the reduction of salt concentration. P_load_ increases and the force balance of the reservoirs is broken. Then, the structural characteristics of porous media changes which cause the porous media deformation or the stacking structure change. At the same time, the cement effect between the porous media particles changes. Then, the migration phenomenon of porous medium particles appears. As a result, the sand-producing phenomenon appears [14,16]. Therefore, the investigation of P_load_ can be favorable for the investigation of the hydrate exploitation of the offshore natural gas hydrate reservoirs.

Xu et al. [22,25] established a fractal model of bentonite swelling, which presented the relationship among void ratio (*e*_m_), P_load_ and *D*. The model is as follows:

(5) $$e_{m}=K\times P_{\mathrm{load}}{}^{D-3}$$

where e_m_ is a ratio of pore volume to porous media volume after the swelling of porous media, which is not related with the initial water content and the dry density of the porous media samples; K is the swelling coefficient of porous media. The sand-bentonite mixture indicated that the constant of the *e*_m_ and P_load_ relationship is independent of the sand content in the bentonite. Therefore, K of the natural sediments samples relates mainly to the content of montmorillonite.

According to Equation (5), we obtain the relationship between K and P_load_ of five natural sediments samples using the above experimental measure results of *e*_m_ and *D*, which show as follows:

(6) $$\mathrm{NSI}:\;P_{\mathrm{load}}{}^{0.51}=1.15\times K$$

(7) $$\mathrm{NSII}:\;P_{\mathrm{load}}{}^{0.50}=0.88\times K$$

(8) $$\mathrm{NSIII}:\;P_{\mathrm{load}}{}^{0.50}=1.19\times K$$

(9) $$\mathrm{NSIV}:\;P_{\mathrm{load}}{}^{0.47}=1.12\times K$$

(10) $$\mathrm{NSV}:\;P_{\mathrm{load}}{}^{0.43}=1.10\times K$$

The results indicate the investigation of the physical characteristics of porous media and the swelling characteristics of porous media can provide a basic date and scientific basis for furthering the mechanical characteristics of reservoir in the hydrate exploitation in the offshore gas hydrate reservoir.

## 4. Conclusions

In this work, we explored the physical characteristics and the swelling characteristics of the human-made porous media and the natural sediments from different sea areas. The effects of the material of porous media, water content, the initial porosity and salinity on swelling characteristics of porous media were discussed. Then, the mechanism of the influence of porous media swelling characteristics on the structural characteristics of the reservoir during the natural gas hydrate dissociation process was tentatively explored. Appealing results and valuable conclusions are found through experiments and calculations:

1. The swelling of porous media composes the primary swelling stage and the secondary swelling stage. In the primary swelling stage, free water fills the space between particles of porous media and the pores of porous media. In the secondary swelling stage, the bound water is absorbed by porous media. Salt ions mainly affect the swelling of bound water in porous media;
2. The swelling rate of porous media is directly proportionate to water content and the initial porosity, and inversely proportionate to salinity. The swelling strain of montmorillonite with the water content of 200 wt% is about twice that of montmorillonite with the water content of 50 wt%. The swelling strain of porous media (montmorillonite) with the initial porosity of 30% is about three times that of montmorillonite with the initial porosity of 60%. The swelling strain of montmorillonite with salinity of 0.2 mol/L is about one and a half times that of montmorillonite with salinity of 1.0 mol/L. Therefore, compared with water content and salinity, the initial porosity has much obvious influence on the swelling;
3. The swelling characteristics of the natural sediments are influenced by the coupling of the montmorillonite content and the salt ion concentration;
4. The influence mechanism of the swelling characteristics of porous media on the structural characteristics of reservoir can be described as: the swelling force of porous media increases due to the salt dilution effect caused by the hydrate dissociation. The force balance of the reservoirs is broken. Then, the structural characteristics of porous media changes which cause the porous media deformation or the stacking structure change.

The swelling characteristics of porous media changes would be one of reasons for the occurrence of sand-producing during the hydrate exploitation. The measurement of the physical property and the swelling of the natural sediments provide the basic date for the further investigation of structural characteristics of the natural sediments and the hydrate dissociation in the natural sediments.

## Figures and Tables

**Table 1:** The place of production of the porous media samples.

Serial Number	Porous Media	Sources	Depths/m
1	montmorillonite	NANOCOR Company, Arlington Heights, IL, USA	—
2	quartz sand (80 mesh)	Shanghai McLean Biochemical Technology Co., Ltd., Shanghai, China	—
3	quartz sand (200 mesh)	Shanghai McLean Biochemical Technology Co., Ltd., Shanghai, China	—
4	kaolinite	Shanghai Aladdin Biochemical Technology Co., Ltd., Shanghai, China	—
5	NSI	Qiong Dongnan Basin	3600
6	NSII	East China Sea	959
7	NSIII	Shenhu Area of South China Sea	1070
8	NSIV	Shenhu Area of South China Sea	1400
9	NSV	Shenhu Area of South China Sea	3415

**Table 2:** Experimental parameters of the swelling characteristics of the porous media samples.

Factors	Parameters			
salinity/(mol·L^−1^)	0.0	0.2	0.5	1
water content/wt%	20	50	100	200
porosity/%	20	30	40	50

**Table 3:** Main components of the porous media samples.

Components	NSI/%	NSII/%	NSIII/%	NSIV/%	NSV/%	Montmorillonite/%	Quartz Sand/%
SiO_2_	29.69	50.11	49.13	42.85	51.90	55.26	99.20
CaO	17.55	13.36	15.47	19.32	8.35	0.42	0.01
Al_2_O_3_	10.50	15.10	13.86	13.5	17.13	19.32	0.52
Fe_2_O_3_	3.37	5.60	5.50	5.34	7.20	1.84	0.05
MgO	2.54	2.73	2.27	2.27	2.90	3.03	0.03
K_2_O	1.48	2.77	2.35	2.23	2.90	0.22	0.08
Na_2_O	1.00	1.68	1.39	1.57	1.96	3.63	0.00
SO_3_	0.72	0.52	0.24	0.29	0.53	0.37	0.00
P_2_O_5_	0.10	0.17	0.17	0.13	0.12	0.02	0.01
MnO	0.05	0.06	0.09	0.34	0.12	0.01	0.00
TiO_2_	0.45	0.80	0.77	0.68	0.81	0.00	0.02
O	31.49	5.33	7.87	9.97	3.91	15.43	0.08
Cl	0.90	1.61	0.68	1.35	2.01	0.13	0.00

**Table 4:** Physical properties of the porous media samples.

Physical Properties	NSI	NSII	NSIII	NSIV	NSV	Montmorillonite	Quartz Sand (80 mesh)	Quartz Sand (200 mesh)	Kaolinite
Mean particle size/μm	9.9	37.8	6.4	3.2	52.4	17.4	262.9	32.6	8.5
Density/(g/cc)	2.1	2.0	2.4	2.5	1.9	2.3	2.1	2.5	2.4
*D*	2.49	2.50	2.50	2.53	2.57	2.40	2.33	2.31	2.51

## Data Availability

Not applicable.
